# Casting a Wider Net: Engaging Community Health Worker Clients and Their Families in Cancer Prevention

**DOI:** 10.5888/pcd13.160114

**Published:** 2016-09-15

**Authors:** Lee Anne Roman, Ruth Enid Zambrana, Sabrina Ford, Cristian Meghea, Karen Patricia Williams

**Affiliations:** Author Affiliations: Lee Anne Roman, Sabrina Ford, Cristian Meghea, Michigan State University, East Lansing, Michigan; Ruth Enid Zambrana, University of Maryland, College Park, Maryland.

## Abstract

Engaging family members in an intervention to prevent breast and cervical cancer can be a way to reach underserved women; however, little is known about whether family member recruitment reaches at-risk women. This study reports the kin relationship and risk characteristics of family members who chose to participate in the Kin Keeper^SM^ cancer prevention intervention, delivered by community health workers (CHWs) via existing community programs. African American, Latina, and Arab family members reported risk factors for inadequate screening, including comorbid health conditions and inadequate breast or cervical cancer literacy. CHW programs can be leveraged to reach underserved families with cancer preventive interventions.

## Objectives

Health-seeking behavior typically occurs in the context of close family relationships outside of clinical and public health settings (1). The Kin Keeper cancer prevention intervention delivers breast and cervical cancer education to underserved women and their family members (2). The program is not stand-alone; the 2-session intervention piggy-backs on existing community health worker (CHW) programs. However, little is known about family member recruitment and whether family networks are a viable approach for reaching at-risk, underserved women (3). This article describes the risk characteristics of family members engaged in the Kin Keeper trial for 3 racial/ethnic groups.

## Methods

An exploratory analysis was conducted using baseline data from the Kin Keeper randomized trial of a population of African American, Latina, and Arab women and their family members (2). The study was conducted in Detroit and Dearborn, Michigan, and delivered through existing CHW programs (eg, a diabetes program) from June 2010 to February 2015. After being trained, CHWs engaged clients of the program, who in turn invited family members to participate in the intervention in their home. At enrollment, participants completed a questionnaire that inquired about sociodemographic characteristics, health status, health care, health literacy, and screening behaviors related to breast and cervical cancer. Cancer literacy was measured with the Breast Cancer Literacy Assessment Tool (4), a 35-item instrument that assesses respondents’ level of knowledge about breast cancer under these these domains: awareness, knowledge and screening, and prevention and control; cervical cancer literacy was assessed with the Cervical Cancer Literacy Assessment Tool (5), a 24-item instrument that assesses the same domains. Adequate cancer literacy was defined as a score of 75% or higher. Appropriately timed screening was defined as 1) clinical breast examination and mammogram (for women aged ≥40 y) in the previous 12 months; and 2) cervical screening in the previous 3 years. Definitions were based on 2007 recommendations of the American Cancer Society (6) and the 2002 US Preventive Services Task Force (USPSTF) for mammography (7) at the time of study design and are consistent with the Affordable Care Act stipulating coverage for mammography based on the 2002 USPSTF recommendations (8). Exposure to cancer media was defined as seeing, reading, or hearing information about breast or cervical cancer prevention.

Bivariate analyses were used to explore differences between the CHW clients and their family members. Significance was set at *P* < .05. The study was approved by the Michigan State University institutional review board.

## Results

Overall, 58% of family members were sisters of the CHW program participants, and more than half were sisters in each of the racial/ethnic groups (Table). Other family participants were mothers (15%), daughters (12%), and aunts (11%). Most family members were aged 40 or older, less than half had any type of employment, 31% had less than a high school education, 23% reported an annual family income of less than $10,000, and 28% lacked public or private health insurance. Almost a quarter (24%) of family participants reported hypertension, 16% reported diabetes, and one-quarter reported tobacco use. Most family members reported little exposure to cancer information from media, and 50% or more had no doctor recommendation for mammography or a Pap test. Most family members had inadequate breast (55%) or cervical (64%) cancer literacy. Only 62% had a mammogram in the previous 12 months, and 72% had a Pap test in the previous 3 years.

**Table:** Bivariate Comparisons of CHW Program Participants to Recruited Family Member Participants, Overall (N = 516) and by Racial/Ethnic Group^a^

Characteristic	All		African American		Latina		Arab	
	CHW Participants (N = 171)	Family Participants (n = 343)	CHW Participants (n = 72)	Family Participants (n = 144)	CHW Participants (n = 22)	Family Participants (n = 43)	CHW Participants (n = 78)	Family Participants (n = 157)
**Demographic**								
**The family member I invited is my . . . b**								
Daughter	11.6	NA	11.2	NA	7.69	NA	13.0	NA
Mother	15.0	NA	9.7	NA	15.38	NA	19.5	NA
Granddaughter	2.4	NA	3.7	NA	0	NA	0.6	NA
Sister	58.4	NA	53.0	NA	66.67	NA	61.0	NA
Aunt	11.3	NA	14.2	NA	5.13	NA	5.2	NA
**Mean age, y**	43	45	42	45	41	43	45	46
<40	33.7	33.0	43.1	35.9	43.86	39.5	22.4	28.5
40–49	37.3	32.7	29.2	28.9	42.86	32.6	43.4	36.4
≥50	29.0	34.2	27.8	35.2	14.29	27.9	34.2	35.1
**Education level <high school**	27.7	31.3	5.8	11.5	61.9	69.0	38.2	39.0
**Single/never married**	25.0	29.7	52.9	50.7	4.55^c^	24.4	5.3	10.9
**Annual family income <$10,000**	26.9	23.0	22.2	20.1	59.09	39.5	22.1	21.2
**Work full-time, work part-time, or self-employed**	46.4	43.3	61.4	61.6	31.82	29.7	36.8	30.3
**No health insurance coverage**	46.1^c^	28.4	29.6	16.1	77.27	53.7	52.7	33.1
**Health**								
High blood pressure	23.4	24.5	37.5	29.2	18.18	14.0	11.7	23.1
Diabetes	31.6^c^	16.0	20.8	15.3	13.64	18.6	46.8^c^	16.0
Depression	13.4	10.2	9.7	9.7	13.64	7.0	16.9	11.5
Tobacco use	24.1	24.8	22.2	31.4	—d	2.3	33.3	25.2
Average, poor, or very poor health	28.6	26.4	21.1	27.3	36.37	27.9	28.4	30.1
**Health Care**								
Difficult access to the health provider	14.7^c^	8.0	12.5	6.3	27.27	16.7	13.2	7.2
Needed to reschedule appointments	41.8	35.5	44.1	29.8	40.91	57.6	39.7	35.2
No doctor recommended clinical breast exam past year	55.4^c^	65.4	64.3	63.4	72.73	65.8	42.1^c^	67.1
No doctor recommended mammography past year (age ≥40)	54.5	49.8	61.0	48.9	66.67	46.2	47.5	51.4
No doctor recommended PAP test past 3 years	56.8	60.2	70.8	60.1	59.09	60.5	42.7^c^	60.3
Clinical breast exam in the past 12 months	63.1	59.8	63.4	60.1	54.55	44.2	65.3	63.8
Mammography in the past 12 months (age ≥40)	63.4	62.2	70.0	59.3	33.33	57.7	62.7	68.2
PAP test in the past 3 years	84.0^c^	71.6	90.3	79.9	90.91	55.8	76.0	68.6
**Health Literacy**								
Inadequate breast cancer literacy	52.0	54.8	54.2	50.7	68.18	69.8	45.4	54.5
Inadequate cervical cancer literacy	60.8	64.4	58.3	60.4	63.64	74.4	62.3	65.4
No knowledge of cancer history in the family	24.7	25.7	27.8	32.6	22.73	32.6	22.4	17.7
No or low recent exposure to breast cancer media	53.3	45.6	44.9	32.6	81.82	61.9	52.6	52.6
No or low recent exposure to cervical cancer media	65.7	60.5	56.3	52.2	95.45	78.0	65.8	63.0

Abbreviations: CHW, community health worker; NA, not applicable; PAP, Papilloma.

a Values are percentages unless otherwise indicated.

b Only the recruited family members (ie, not the initial CHW program participant) responded to this question.

c *P* < .05.

d Number for Latinas who used tobacco was too small to calculate.

Compared with the CHW program’s clients, family members were less likely to have no health insurance (28% vs 46%), were less likely to have diabetes (16% vs 32%), and have less difficulty accessing a health care provider (8% vs 15%); however, they were also less likely than CHW clients to have had a Pap test in the past 3 years (72% vs 84%). Among African American women, there were no differences in demographic, health, or health care access characteristics or in cancer screening and literacy between the family participants and the CHW clients. This was also true for Latinas, with one exception: family participants were more likely to be single or never married than CHW clients (24% vs 5%). Among Arab women, family participants were less likely to have diabetes (16% vs 47%) and more likely to have their doctor not recommend a clinical breast exam in the previous year (67% vs 42%) or a Pap test (60% vs 43%) than were CHW clients.

## Discussion

CHW programs that focus on chronic illness, wellness, or other issues and that enroll underserved African American, Latina, or Arab women were successful in engaging their clients recruit family members for a preventive intervention. Most CHW clients invited their sisters, and most women were at midlife, an age when it is especially important to intervene to prevent cancer later in life (9). Family members had risk factors for inadequate screening, including financial stressors, comorbid health conditions, and lack of health insurance (10). Most family members and CHW clients had inadequate breast and cervical cancer literacy and low exposure to cancer media. Although family members were more likely to be insured, cancer screening rates among family members were similar to (breast) or lower than (cervical) rates for clients. Therefore, CHW clients could be important health advocates for other family members. Although the CHW clients were more likely to be uninsured, this finding may be a function of how community-based CHW programs engage uninsured women. Family members were less likely than CHW clients to have diabetes; however, some clients were invited through a diabetes program. A limitation of the study is that characteristics and screening participation data were self-reported.

To address persistent cancer inequalities, there are calls for increased attention to multilevel factors, such as family context, to improve cancer care and outcomes (11). When family members participate together in an intervention, it allows for shared understanding with the potential that family members will reinforce preventive health behavior for each other. CHWs that serve individual clients can be an important bridge to families (12), and community programs can be leveraged to reach underserved, at-risk women within the broader family unit. To deliver family-focused services, partnerships across public health programs, integration of cancer literacy and screening education, and cross-training of CHWs are needed.
